# Biomechanical Responses to Level, Uphill, and Downhill Running Segments During an Outdoor Marathon

**DOI:** 10.1002/ejsc.70261

**Published:** 2026-10-05

**Authors:** Ransi S. S. Subasinghe Arachchige, Ke Hu, Yannis Pitsiladis, Carl James, Roy T. H. Cheung

**Affiliations:** ^1^ Human Movement Laboratory School of Health Sciences Western Sydney University Sydney New South Wales Australia; ^2^ Department of Sports and Health Sciences Faculty of Arts and Social Sciences Academy of Wellness and Human Development Hong Kong Baptist University Hong Kong China; ^3^ Centre for Exercise Science and Medicine (CESAME) Hong Kong Baptist University Hong Kong China; ^4^ Department of Biology Faculty of Science Hong Kong Baptist University Hong Kong China; ^5^ Human Telemetrics Ltd London UK; ^6^ Translational Health Research Institute Western Sydney University Sydney New South Wales Australia

**Keywords:** loading metrics, running biomechanics, slope, velocity, wearable sensors

## Abstract

This study investigated running biomechanics across varying slopes during a mass‐participation road marathon. Although laboratory studies suggest specific gait modifications occur in response to varying slopes, data from outdoor competitive environments remain scarce. We monitored 39 distance runners using shoe‐mounted inertial measurement units to capture spatiotemporal, footstrike, and wearable‐derived loading data across level, uphill (+3%), and downhill (−3%) segments of the 2025 Hong Kong Marathon. Results demonstrated slope‐dependent modifications, with uphill running characterized by slower velocity (−0.29 m/s, Cohen's *d* = −1.98), shorter stride length (−0.15 m, *d* = −1.55), and lower cadence (−5.00 steps/min, *d* = −1.21) compared with level ground (*p* < 0.001). Kinematically, participants adopted a more anterior footstrike pattern uphill (*d* = 1.48) and a more posterior footstrike pattern downhill (*d* = −0.62) relative to level running (*p* < 0.05). Analysis of wearable‐derived loading metrics showed no differences across slope conditions once running velocity was included as a covariate. Running velocity emerged as the primary determinant of braking, impact, and shock accelerations, indicating that differences in self‐selected running velocity may contribute substantially to loading responses across varying slopes during overground running. These findings highlight a discrepancy between controlled laboratory simulations and competitive overground running, emphasizing the role of wearable technology in capturing ecologically valid gait alterations to different terrains.

## Highlights

- Wearable IMUs captured ecologically valid biomechanics during a mass‐participation marathon, revealing gait adaptations across level, uphill, and downhill terrain beyond what laboratory studies can replicate.
- Uphill running showed clear spatiotemporal and footstrike changes, whereas downhill segments displayed limited biomechanical differences, indicating slope‐specific strategies shaped by self‐selected pacing.
- Loading metrics did not differ across slopes once velocity was controlled, indicating that running velocity was associated with braking, impact, and shock demands rather than slope per se, although the direction of this relationship cannot be established from the present observational data.
- Findings challenge laboratory assumptions about downhill loading, emphasizing the value of wearable technology for studying real‐world gait behaviour and informing coaching and injury‐prevention practice.

## 1 Introduction

Overground running biomechanics are strongly influenced by environmental factors, with terrain slope shaping mechanical demands that affect both performance and injury prevention (Lemire et al. 2022). Both uphill and downhill running display mechanical adjustments, including modified stride, foot placement, and loading, as athletes seek to maintain efficiency and reduce musculoskeletal stress (Gottschall and Kram 2005; Vernillo et al. 2017).

Compared with level running, uphill running (+2–15%) is associated with spatiotemporal and kinematic adaptations, such as increased cadence (approx. +3–7%) and a shift toward mid‐to forefoot strike patterns (Vernillo et al. 2017), which may reflect both propulsion demands and the mechanical constraints of running on steep gradients. Additionally, impact forces are lower, with reduced peak ground reaction forces but increased propulsive demands (Gottschall and Kram 2005). In contrast, downhill running typically displays a lower cadence and a tendency to adopt a rearfoot strike pattern (Lu et al. 2025). Similar slope‐related alterations in step mechanics and touchdown posture have also been observed during sprint running, reinforcing that gradient fundamentally modifies lower‐limb kinematics across running modalities (Paradisis and Cooke 2001). Previous biomechanics studies demonstrated that slope fundamentally alters running kinetics (Vernillo et al. 2017, 2020), which could pose a greater risk for running‐related injuries (Chan et al. 2018).

However, these alterations have largely been observed in laboratory settings, raising concerns about their generalizability to overground running. In a field‐based study involving 20 trail runners, changes in spatiotemporal parameters did not align with those found in previous laboratory‐based investigations (Genitrini et al. 2023). Although the study explored differences during uphill and downhill sections between more proficient and less proficient runners, qualitative interpretation of the published plots indicated consistent distinctions between uphill and downhill slopes. There was a decrease in both cadence and step length during uphill running (+12% slope), compared with downhill running (−12% slope), regardless of experience level (Genitrini et al. 2023). This contradicts laboratory‐based studies reporting decreased cadence (−8% slope; approx. −4% cadence) (Lussiana et al. 2013) and stride length in downhill running (−17.6% slope; −4% stride length) (Devita et al. 2008) when compared with uphill running, highlighting the need for further investigation into slope‐related biomechanical responses during outdoor competitive running. Furthermore, to isolate the effect of slope on kinetic running measures, most studies have examined changes in running biomechanics using laboratory‐simulated slopes at a fixed test speed (Gimenez et al. 2014; Vernillo et al. 2017, 2020). However, such methodologies fail to capture the natural variability of outdoor running, where self‐regulated pacing and terrain fluctuations can introduce meaningful differences (DeJong Lempke et al. 2025).

Recent advances in wearable technology now allow researchers to address these limitations by capturing biomechanical data in real‐world running environments (DeJong and Hertel 2020; Koldenhoven and Hertel 2018). Large‐scale field studies have demonstrated how inertial sensors attached to athletes' footwear can monitor changes in biomechanical responses during competition, such as the Adidas Road to Records (Guppy et al. 2023) and World Triathlon Cup (James, Muniz‐Pardos, et al. 2025) as well as across different footwear conditions (Muniz‐Pardos et al. 2025). Commercially available IMUs can provide a series of metrics, including cadence, footstrike patterns, impact, peak ground reaction forces, and braking forces, with high validity outside laboratory settings (Zeng et al. 2022). Prior work has shown that these wearable‐derived features are sensitive to temporal changes during running, with small‐to‐moderate increases in impact and braking metrics and modest alterations in cadence and foot strike characteristics observed across a 5‐km run, supporting their utility for detecting fatigue‐related biomechanical adaptations (DeJong Lempke et al. 2025).

Footstrike modifications to changes in slope and running velocity are scarcely reported outside laboratory protocols, with few studies assessing how slope modulates the relationship between footstrike pattern, braking impulse, and vertical loading rate in real‐world conditions. Although an observational study by Vermand et al. (2022) focused on within‐slope comparisons to characterize fatigue‐related changes during a 40‐km mountain race, their descriptive dataset highlights differences between uphill, flat, and downhill segments (Vermand et al. 2022). Uphill running was characterised by a higher proportion of forefoot striking compared with flat and downhill segments across both halves of the race, whereas cadence remained relatively consistent and impact‐related metrics showed modest increases. Work by Giandolini et al. (2017), conducted in an outdoor trail race, reinforced that footstrike pattern substantially influences neuromuscular demand, with forefoot striking eliciting greater plantarflexor activation and fatigue than rearfoot striking (Giandolini et al. 2017). Collectively, these findings highlight a gap in understanding how slope, velocity, and footstrike interact to influence loading mechanics during outdoor running.

This study investigated the effect of slope (i.e., uphill, level and downhill) on spatiotemporal, kinematic, and wearable‐derived loading metrics during a mass‐participation road marathon. We hypothesized that participants would demonstrate slope‐specific modifications, with uphill running associated with decreased velocity and greater prevalence of forefoot and midfoot strikes, along with reduced braking impulse and impact acceleration. Conversely, downhill running was hypothesized to show increased velocity, a predominant rearfoot strike pattern, greater braking impulse, and higher impact.

## 2 Materials and Methods

### 2.1 Participants

The study cohort consisted of 39 distance runners recruited from the 2025 Standard Chartered Hong Kong Marathon and Half‐Marathon (Table 1), encompassing a broad spectrum of runners from amateur distance athletes to experienced international competitors in their respective categories. Participants were aged between 15 and 65 years and, as registered entrants in the marathon event, were assumed to be in adequate health and training condition to complete their respective races. Participants were excluded if they reported a current or recent musculoskeletal injury within the preceding 3 months that limited training or competition, a history of lower‐limb surgery, or a confirmed cardiovascular, neurological, or metabolic condition contraindicating endurance exercise. Half‐marathon runners averaged 36.4 ± 9.9 km/week across 2.8 ± 1.1 sessions, whereas marathon runners averaged 56.8 ± 8.8 km/week across 3.6 ± 1.2 sessions. Weekly training distance differed between groups (*p* < 0.001). Participants completed their respective running events without receiving any specific instructions or pacing advice. All runners used their own footwear during the event.

**TABLE 1:** Participant characteristics.

Demographics	Half‐marathon (*n* = 22)	Marathon (*n* = 17)	*p*
Sex (male/female)	13/9	13/4	0.254
Age (years)	39.4 ± 13.7	40.5 ± 13.3	0.792
Height (m)	1.69 ± 0.10	1.68 ± 0.10	0.805
Mass (kg)	63.00 ± 9.91	62.70 ± 10.60	0.943
Body mass index (kg/m^2^)	22.00 ± 2.34	22.10 ± 2.26	0.896

*Note:* Data reported as mean ± standard deviation.

The sample size required for this study was determined using G*Power (Version 3.1, University of Kiel, Germany), informed by prior work demonstrating slope‐related differences in temporo‐spatial running parameters across mild slopes (Padulo, Annino, Migliaccio, et al. 2012). Using the reported means and standard deviations for step frequency and step length, we calculated approximately large effect sizes for between‐slope differences across both elite and amateur marathon runners. However, because the present study examined multiple biomechanical parameters and was conducted in real‐world outdoor running conditions, we used a conservative moderate effect size (Cohen's *f* = 0.25). Assuming *α* = 0.05, power = 0.8, and a correlation of 0.5 among the three repeated slope measurements, a total of 28 participants was calculated to be sufficient for this study. The correlation of 0.5 represents G*Power's default setting for repeated measures and was selected as a widely accepted and recommended practice in the absence of a known correlation from similar previous studies. This estimate served as a pragmatic guide, as formal a priori power calculations for mixed‐effects models with covariates are not well established. All experimental procedures were reviewed and approved by the Hong Kong Baptist University Research Ethics Committee (REC/23‐24/0028/A5), and written informed consent was provided by each participant. For athletes aged 15 to 18 years, parental consent was obtained in accordance with the approved protocol permitting non‐invasive participation.

### 2.2 Running Course

Of the 39 participants, 22 competed in the half‐marathon (21.0975 km) and 17 in the marathon (42.195 km). Both courses were point‐to‐point incorporating major bridges, tunnels, and highway segments (Standard Chartered Hong Kong Marathon 2025). The marathon course featured 254 m of ascent and 243 m of descent (net gain: 11 m), whereas the half‐marathon had 190 m of ascent and 180 m of descent (net gain: 10 m), as shown in Figure 1. The half‐ and full‐marathon courses used a shared route, with the marathon having an additional 21.1 km between approximately 4–26 km. Extracted data for flat, uphill and downhill segments occurred at identical locations.

**FIGURE 1:**
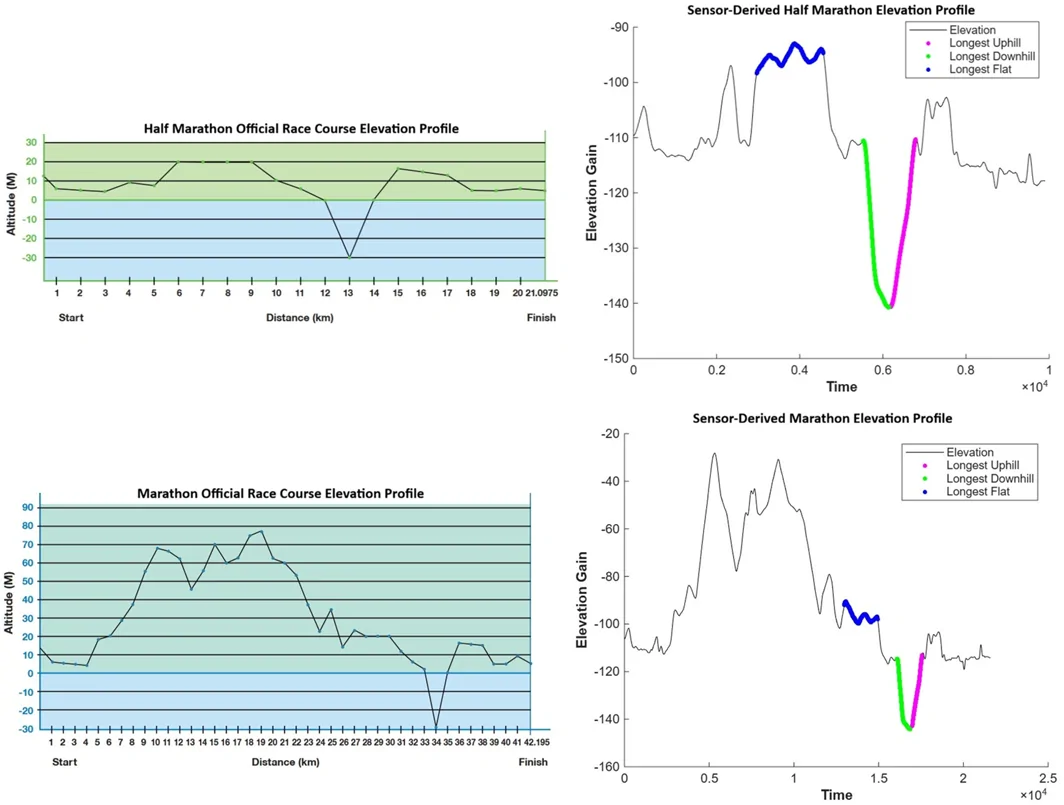
Comparison of elevation profiles for the half and full marathons. Left panel: elevation profile derived from participant RunScribe data processed in MATLAB. The *y*‐axis reflects the relative elevation change in meters from the device baseline. Right panel: Official elevation profile extracted from the race organizer’s guide.

### 2.3 Instrumentation

A RunScribe Plus unit (Scribe labs, Moss Beach, California, United States) was attached to the shoelaces of the right shoe for each participant. The device consisted of a tri‐axial IMU sampling at 500 Hz, enabling the measurement of spatiotemporal, footstrike, and wearable‐derived loading parameters. Spatiotemporal parameters obtained from RunScribe have previously shown *good* to *excellent* agreement (ICC > 0.73) with motion‐capture systems (Koldenhoven and Hertel 2018). RunScribe classifies footstrike on a continuous scale from 1 to 16, where lower values (i.e., 1–5) indicate a more rearfoot‐dominant strike pattern, and higher values (i.e., 11–16) suggest a more forefoot‐dominant strike. Values near the midpoint (i.e., 6–10) reflect a midfoot strike (RunScribe 2016). This measurement has also been validated against laboratory measurement of footstrike angle (DeJong and Hertel 2020), while shock, impact, and braking acceleration also show strong agreement (ICC > 0.89) (Brayne et al. 2018). However, as these reliability estimates were derived from different samples and settings, they do not substitute for reliability analysis specific to the present outdoor, fatigued, self‐paced context.

### 2.4 Data Processing

Data were trimmed to the event length using the RunScribe website before being downloaded and exported into a CSV file. MATLAB (version R2023b, The MathWorks Inc., Natick, MA, United States) was used to process and analyse all data. The slope was calculated by differentiating the elevation signal and smoothed using a 70‐point moving average, corresponding to approximately 0.14 s of data at the 500 Hz sampling rate. This window was chosen to reduce high‐frequency noise in the elevation signal while still preserving terrain changes. Each data point was classified into one of three surface slope conditions based on the following thresholds: uphill (slope > 0.035), downhill (slope < −0.035), or flat (|slope| ≤ 0.035). This threshold is equivalent to approximately 2° and was chosen to minimize misclassification arising from small fluctuations in the elevation signal. The longest continuous segment of each slope type was then identified for subsequent analysis, with uphill and downhill segments approximately 1 km in length, and the flat section spanned approximately 3 km (Figure 1). Although outdoor terrain varies continuously, the slope was categorized into uphill, level, and downhill to enable consistent comparison across participants. Selecting the longest uninterrupted segment ensured that each slope condition reflected steady‐state running rather than transient adjustments during short transitions where rapid changes in gradient or pacing could confound classification. Using multiple shorter segments was avoided because many runners encountered fragmented slope exposures of unequal duration, which would have reduced comparability across participants. Flat, uphill and downhill segments occurred at identical locations of the half‐marathon and marathon routes. This corresponded to relative race distances of 28%, 62% and 57% for the level, uphill, and downhill segments of the half‐marathon, respectively. For the full marathon, these segments occurred at 64% (level), 81% (uphill) and 78% (downhill) of the race distance.

The steepest uphill and downhill sections corresponded to slopes of approximately +3% and −3%, respectively. The indices corresponding to the identified slope segments were used to calculate mean values of the spatiotemporal, footstrike, and wearable‐derived loading parameters being analysed. For loading metrics, we included impact, braking, and shock, which corresponded to the vertical component of peak acceleration, the horizontal component of peak deceleration, and the resultant metric integrating both impact and braking, respectively. To ensure that only running strides were included in the analysis, participants whose mean velocity across the identified slope segments fell below a threshold velocity of 1.8 m/s were excluded, reflecting the lower bound of the preferred walk–run transition velocity (1.9–2.1 m/s) (Gunderson 2011; Rotstein et al. 2005). Using 1.8 m/s therefore provides a conservative cutoff for distinguishing running from walking, particularly under conditions of fatigue and mild inclines (Vermand et al. 2022). After applying the 1.8 m/s threshold, no participants or slope segments met the exclusion criteria, and all 39 participants contributed data to each slope condition.

### 2.5 Statistical Analysis

All analyses were performed using IBM SPSS Version 29 (IBM Corp., Armonk, NY, United States). Baseline differences between race groups were evaluated using independent‐sample *t*‐tests for continuous variables and a chi‐square test for sex distribution. Data normality was assessed using the Shapiro–Wilk test. A linear mixed‐effects model was conducted to examine the effects of slope condition on running velocity, stride length, cadence, footstrike pattern, braking, impact, and shock. Participants were included as a random intercept to account for repeated measures, while slope (uphill, flat, downhill) and race type (half‐marathon, marathon) were included as fixed effects. Race type was included as a fixed effect to account for the fact that the identified slope segments occurred at different relative distances in the half‐marathon and marathon events, ensuring that slope‐related effects were estimated after adjusting for event distance. For braking, impact and shock, running velocity was added as a covariate to account for its known influence on loading metrics (Brughelli et al. 2011) and adjusted pairwise slope contrasts with 95% confidence intervals were reported. Where significant main effects were observed, post hoc pairwise comparisons with Bonferroni adjustment were performed. The significance level was set at 0.05. Cohen's *d* was used to quantify effect sizes and reduce overreliance on *p*‐values, with values of 0.2, 0.5, and 0.8 considered small, medium, and large effects, respectively (Cohen 2013) and computed using a repeated‐measures formulation appropriate for within‐participant comparisons.

## 3 Results

Mean marathon and half‐marathon completion times were 248 ± 28 and 116 ± 17 min, respectively. A linear mixed‐effects model revealed the effects of slope on fundamental running spatiotemporal parameters (Figure 2). For running velocity, effects of both slope (F_2, 76_ = 48.18, *p* < 0.001), and race type (F_1, 37_ = 9.88, *p* = 0.003) were observed. After adjusting for race type, post hoc comparisons showed that velocity was slower during uphill running compared with level (*p* < 0.001; Cohen's *d* = −1.98) and downhill running (*p* < 0.001; Cohen's *d* = −1.90). There were effects of both slope (F_2, 76_ = 30.44, *p* < 0.001), and race type (F_1, 37_ = 9.32, *p* = 0.004) for stride length. Controlling for race type, post hoc comparisons indicated shortened stride length during uphill running compared with level (*p* < 0.001; Cohen's *d* = −1.55) and downhill conditions (*p* < 0.001; Cohen's *d* = −1.44). Only effects of slope (F_2, 76_ = 18.32, *p* < 0.001) were observed for cadence, demonstrating lower cadence during uphill versus level (*p* < 0.001; Cohen's *d* = −1.21) and downhill running (*p* < 0.001; Cohen's *d* = −1.17).

**FIGURE 2:**
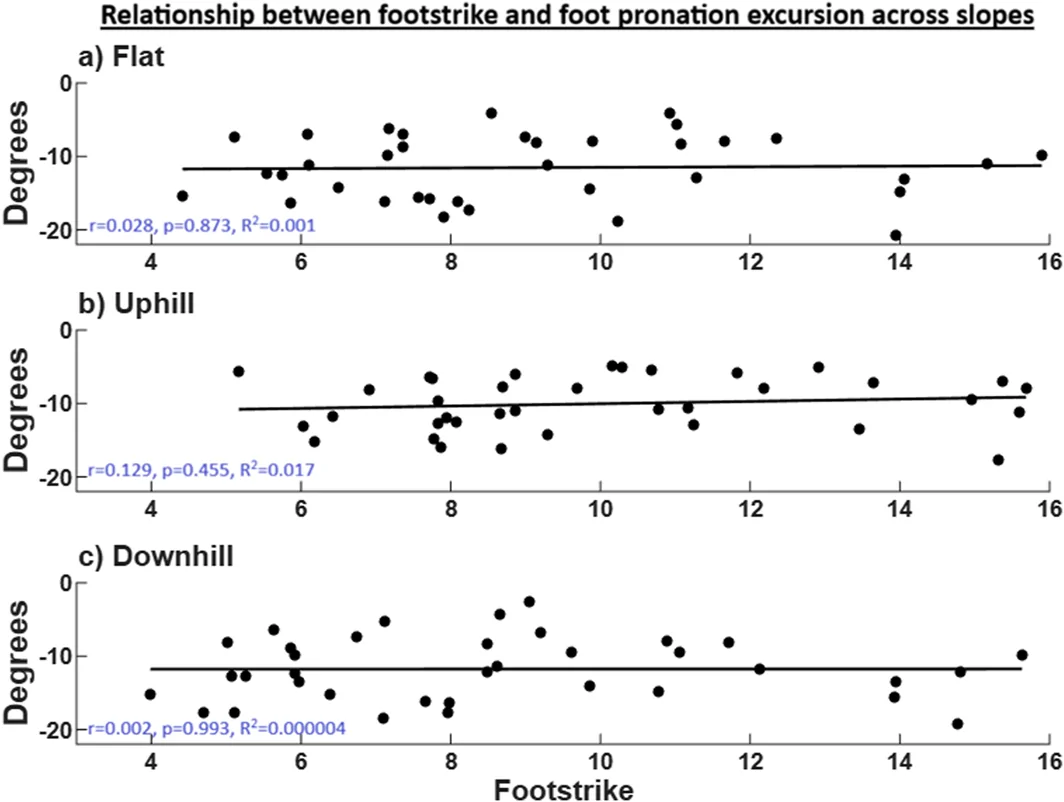
Effects of slope on spatiotemporal parameters. Violin plots illustrate the changes in (a) running velocity (m/s), (b) stride length (m), and (c) cadence (steps/min) across uphill, level, and downhill running conditions. Asterisks indicate a significant difference between conditions (*p* < 0.05). The violin width reflects the distribution of unadjusted data, the central bold line denotes the median, and individual subject trajectories are shown as connecting lines to illustrate within‐subject variation.

Footstrike patterns differed across slope conditions (F_2, 76_ = 45.39, *p* < 0.001, Figure 3). Post hoc comparisons revealed more forefoot striking during uphill running compared with level (*p* < 0.001; Cohen's *d* = 1.48) and downhill running (*p* < 0.001; Cohen's *d* = 2.10). A more rearfoot strike was also found during downhill running versus level ground (*p* = 0.023; Cohen's *d* = −0.62).

**FIGURE 3:**
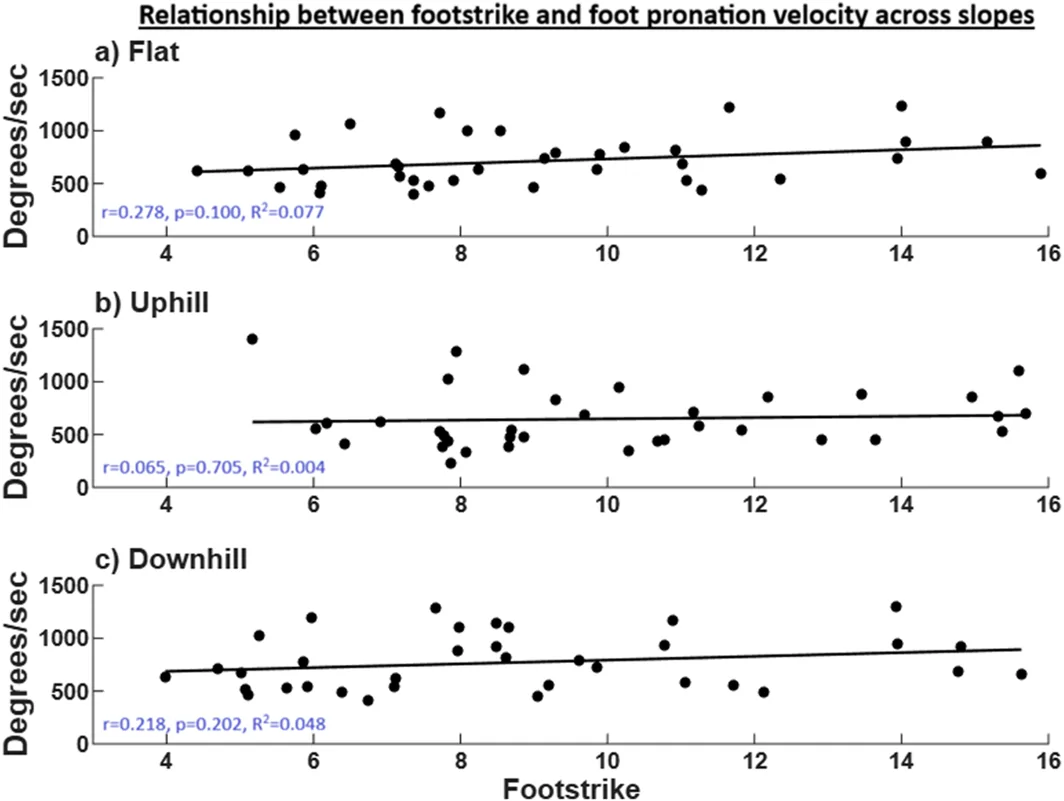
Effects of slope on footstrike pattern. Violin plots illustrate the changes in footstrike index, from 1 (rearfoot) to 16 (forefoot) across uphill, level, and downhill running conditions. Asterisks indicate a significant difference between conditions (*p* < 0.05). The violin width reflects the distribution of unadjusted data, the central bold line denotes the median, and individual subject trajectories are shown as connecting lines to illustrate within‐subject variation.

Loading metrics showed no detectable differences across slope conditions after adjusting for running velocity (Figure 4). For braking, the mixed‐effects model showed no effect of slope (F_2, 86.36_ = 0.46, *p* = 0.634), while running velocity showed an effect (F_1, 111.72_ = 14.90, *p* < 0.001). Adjusted pairwise contrasts were small, with confidence intervals crossing zero (uphill‐downhill: 0.021 g, 95% CI −0.437 to 0.478; uphill‐flat: 0.145 g, 95% CI −0.321 to 0.610; downhill‐flat: 0.124 g, 95% CI −0.236 to 0.484). Similarly, impact acceleration showed no effect of slope (F_2, 87.13_ = 0.26, *p* = 0.776), with velocity again emerging as a covariate (F_1, 111.81_ = 5.80, *p* = 0.018). Confidence intervals again included zero (uphill‐downhill: −0.221 g, 95% CI −1.017 to 0.574; uphill‐flat: −0.209 g, 95% CI −1.017 to 0.599; downhill‐flat: 0.012 g, 95% CI −0.621 to 0.645). Shock acceleration also did not differ across slope conditions (F_2, 89.43_ = 0.38, *p* = 0.685), whereas running velocity showed an effect (F_1, 105.63_ = 20.31, *p* < 0.001). Adjusted contrasts again crossed zero (uphill‐downhill: −0.210 g, 95% CI −0.866 to 0.446; uphill‐flat: −0.066 g, 95% CI −0.731 to 0.600; downhill‐flat: 0.145 g, 95% CI −0.393 to 0.682). The width of these confidence intervals indicates that the present design could rule out slope effects exceeding approximately 0.7 g for shock, 0.8 g for impact acceleration, and 0.5 g for braking, representing a small‐to‐moderate fraction of the raw within‐condition magnitudes of approximately 13–15 g for shock, 8–10 g for impact, and 4–6 g for braking.

**FIGURE 4:**
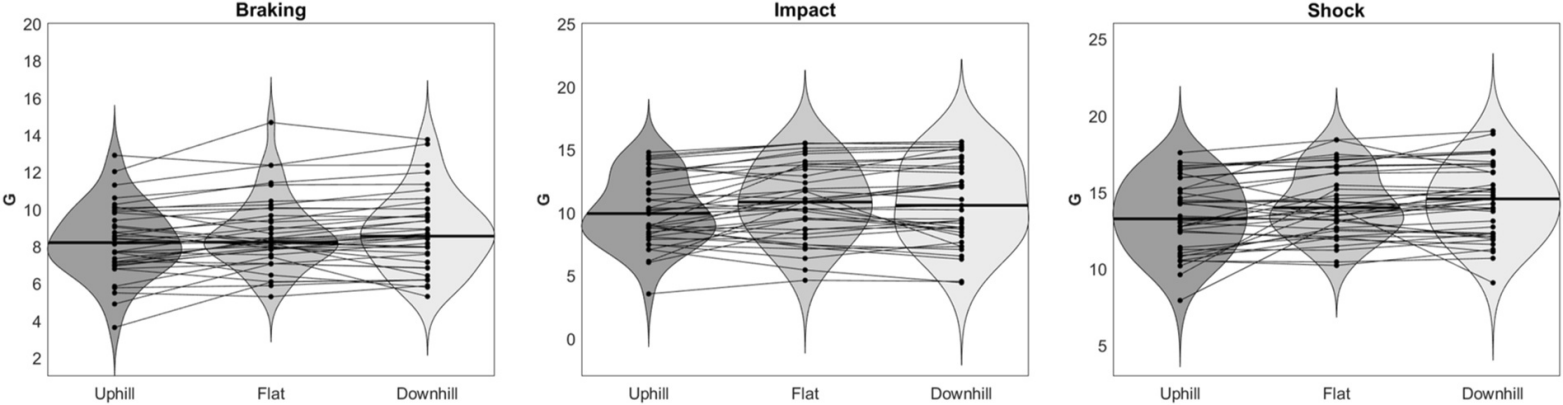
Effects of slope on wearable‐derived loading parameters. Violin plots illustrate the changes in (a) braking (g‐force), (b) impact (g‐force), and (c) shock (g‐force) across uphill, level, and downhill running conditions. The violin width reflects the distribution of unadjusted data, the central bold line denotes the median, and individual subject trajectories are shown as connecting lines to illustrate within‐subject variation.

## 4 Discussion

This study investigated how surface slope influences spatiotemporal parameters, footstrike patterns, and loading responses during an outdoor marathon event. Unlike laboratory studies that constrain speed and isolate slope under controlled conditions, this study captures how runners naturally regulate in response to slope during a real marathon. This provides insight into slope‐specific strategies under conditions of fatigue, self‐selected pacing, and environmental variability that cannot be replicated in laboratory protocols. The findings demonstrate that although uphill running was associated with biomechanical shifts (such as slower velocity, shorter stride length, lower cadence and a greater shift towards a more anterior footstrike pattern), many parameters did not differ between flat and downhill surfaces. Only footstrike patterns showed differences across all three conditions. Interestingly, wearable‐derived loading metrics showed no detectable slope‐related differences once running velocity was accounted for. Our results contrast with common laboratory findings where downhill running typically facilitates higher speeds (Bontemps et al. 2020) and increased loading (Vernillo et al. 2017). The similarity observed between the level and downhill segments of the marathon may reflect the combined influence of self‐selected pacing, accumulated fatigue, physiological capacity, mechanical constraints, and stability demands during prolonged endurance running. Although gravitational assistance might be expected to facilitate faster running downhill, the absence of a higher downhill running velocity in the present study cannot be interpreted as evidence that runners deliberately regulated their speed to maintain biomechanical consistency. It is also possible that physiological or mechanical constraints limited the runners' ability to increase their velocity on the downhill sections. As the present observational design cannot distinguish between these potential mechanisms, the reasons underlying the comparable velocities between level and downhill running require further investigation.

The observed spatiotemporal changes during uphill running align with established theory regarding slower speeds and shorter stride lengths (Padulo, Annino, et al. 2012). However, the observed reduction in cadence during uphill running (−5.00 steps/min vs. flat; −4.90 steps/min vs. downhill) contrasts with several laboratory protocols that display higher cadence (+3–7%) (Vernillo et al. 2017). This divergence may reflect the ecological nature of our data collection, which allowed runners to self‐select running velocity at all times, whereas laboratory studies typically constrain velocity, forcing runners to maintain a fixed speed that necessitates a higher cadence to compensate for shorter strides. Because running velocity was unconstrained in this outdoor marathon event, the concurrent reductions in velocity, cadence, and stride length during uphill segments reflect coupled spatiotemporal adjustments (Padulo et al. 2023). During a marathon, participants continuously regulate their velocity through adjustments to cardiovascular, neuromuscular, and metabolic demands, while also accounting for perceived exertion, thermoregulation, terrain, and tactical considerations (Sha et al. 2024). Continuous IMU‐based monitoring across flat surfaces during a full marathon has shown that runners exhibit progressive, non‐linear fatigue‐related changes, including reductions in velocity, stride length, flight time, and leg stiffness, as the race progresses, reinforcing that self‐selected pacing and race progression strongly shape real‐world running mechanics (Meyer et al. 2021). Findings from a simulated 10‐km race show that fatigability is accompanied by increased gait variability despite relatively stable mean spatiotemporal metrics, indicating that fatigue induces coordinated adjustments in neuromuscular output during continuous outdoor running (Padulo et al. 2025). In this study's outdoor marathon context, cadence may reflect adjustments related to cardiovascular and neuromuscular demands rather than the maintenance of mechanically optimal stride characteristics (Grivas 2025). Maintaining or increasing cadence on an incline substantially elevates muscle activation frequency and metabolic cost (Padulo et al. 2013). Therefore, in a prolonged endurance event, runners may self‐select a lower cadence in response to immediate slope demands. However, it is important to acknowledge that in the present study, slope and race stage were inherently confounded, as the level, uphill, and downhill segments occurred at different relative distances in both the half‐marathon and marathon events. This prevents the separation of slope‐related effects from the influence of race progression, pacing strategy, or accumulated fatigue, and the fatigue narrative cannot be substantiated from the present dataset. These underlying mechanisms should be explored in future studies designed to disentangle slope effects from race progression.

Footstrike pattern was the only metric to show a significant difference across all surfaces, shifting from a more anterior footstrike pattern uphill (+1.3) to a more posterior footstrike pattern downhill (−0.5). A more anterior footstrike during uphill running may facilitate propulsion by increasing the contribution of the ankle plantarflexors and improving the capacity to generate upward impulse (Liebl et al. 2014). Conversely, the shift toward a more rearfoot dominant strike downhill, even when running velocity and loading metrics remained stable, likely served as a primary mechanism for stability and braking control. Slope‐related postural constraints further limit the feasibility of rearfoot striking uphill and forefoot striking downhill, as changes in trunk and lower‐limb orientation mechanically bias foot placement at ground contact (Paradisis and Cooke 2001). This modulation may reflect adjustments in surface interaction and stability demands to manage changing terrain although the underlying neuromuscular mechanisms remain speculative without direct muscle‐level measurements.

Wearable‐derived loading responses showed no detectable slope‐related differences once running velocity was accounted for, with the confidence intervals indicating that the design could rule out slope effects exceeding approximately 0.7 g for shock, 0.8 g for impact, and 0.5 g for braking–values that represent a small‐to‐moderate fraction of the raw within‐condition magnitudes. Importantly, applying a Benjamini–Hochberg correction across the seven primary omnibus tests did not alter any conclusions, with velocity, stride length, cadence, and footstrike remaining significant and braking, impact, and shock remaining non‐significant. This contrasts with laboratory studies reporting increased impact and braking forces during downhill running (Vernillo et al. 2017). Our findings demonstrate that running velocity changed across slope conditions, whereas braking, impact, and shock were associated with running velocity rather than slope per se. The mechanisms underlying these relationships cannot be determined from the present observational data. Factors such as fatigue, physiological demands, self‐selected pacing, and stability requirements may contribute to the observed changes in running velocity and loading responses, but these remain plausible explanations rather than mechanisms directly demonstrated by the present study. The absence of detectable slope‐specific loading differences after accounting for velocity nevertheless highlights the importance of considering velocity when interpreting wearable‐derived loading metrics in real‐world endurance events.

These findings have potential practical relevance for understanding how runners manage mechanical demands across slopes during endurance events. While the slopes examined were moderate, their repeated application over marathon distances may increase cumulative mechanical demands on tissues (Smyth et al. 2022). The observed shift toward a more anterior footstrike pattern uphill and a more posterior pattern downhill likely reflects adjustments related to stability and propulsion demands. These patterns suggest possible trade‐offs between performance and mechanical demands across slopes, supporting practical strategies such as downhill specific conditioning (Shaw et al. 2018) and eccentric strength development (Satkunskienė et al. 2015), although further investigation with physiological and musculoskeletal measures is warranted. We emphasize that our cross‐sectional design does not permit causal inferences regarding injury risk, and any clinical applications would require prospective studies linking these biomechanical patterns to injury outcomes.

A major strength of this study is the ecological design, capturing biomechanical parameters during an outdoor endurance event where factors such as fatigue, self‐selected pacing, varied topography and changing environmental conditions (James, Verdoukas, et al. 2025) are present. This addresses a key limitation of laboratory research where controlled conditions may limit generalizability. Additionally, the simultaneous analysis of spatiotemporal, footstrike, and loading metrics allows for an integrated interpretation of adaptations. Nevertheless, several limitations must be noted. Slope and race stage were inherently confounded, as uphill, downhill, and flat segments occurred at different relative distances in both the half‐marathon and marathon events, preventing the separation of slope‐related effects from accumulated fatigue, pacing strategy, or race progression. Although race type was included as a fixed effect, the design does not isolate pure slope effects. This confounding finding is particularly relevant for the half‐marathon, where the level segment occurred at 28% of the race distance, while uphill and downhill segments occurred at 57% and 62%, respectively, meaning that interpretations invoking fatigue as a primary driver of observed differences cannot be substantiated from the present dataset. Incline sequences could not be controlled, as this limitation is inherent to real‐world race settings where slope order cannot be manipulated or randomized. However, previous field‐based studies have reported similar trends to those observed in the current study, irrespective of slope sequence or fatigue (Genitrini et al. 2023; Vermand et al. 2022). The event featured relatively mild slopes, which may constrain the magnitude of biomechanical adaptations and restrict generalizability to steeper terrain. Weekly training volume differed significantly between the half‐ and full‐marathon groups, which may introduce variability in the observed biomechanical patterns. Slope classification relied on processed elevation data, which may introduce errors during short transitions. However, alignment was cross‐checked against the raw elevation trace and the official course elevation profile. Environmental factors such as crowd density (De Freitas et al. 1985) and surface texture were not controlled. Additionally, the race setting introduced natural differences in footwear, which could not be standardized. A further methodological consideration is the absence of test‐retest reliability analyses for the spatiotemporal, footstrike, and loading metrics within this specific sample. Reliability estimates from prior studies conducted under different conditions, such as controlled laboratory settings and non‐fatigued participants, do not guarantee equivalent precision in the present outdoor, fatigued, self‐paced context. We therefore caution that the precision of our estimates may be somewhat lower than that reported in previous validation studies, and future work should explicitly quantify reliability within similar real‐world endurance settings. Finally, while within‐subject comparisons were used, the study was not designed to link these patterns directly to prospective injury outcomes. Despite these limitations, the use of wearable IMUs during a large outdoor road race provides strong ecological validity by capturing biomechanical responses under conditions that cannot be replicated in laboratory settings. Future work involving more diverse participant samples and complementary physiological or musculoskeletal measures may help validate and extend these findings.

## 5 Conclusion

This study observed distinct slope‐dependent responses in spatiotemporal parameters and footstrike patterns, whereas wearable‐derived loading metrics were primarily influenced by running velocity, rather than slope. Uphill running was characterized by slower velocity, shorter stride length, lower cadence, and a more anterior footstrike pattern. In contrast, downhill running was associated with a more posterior footstrike pattern, but wearable‐derived loading parameters showed no detectable differences across slope conditions once running velocity was controlled for. These findings suggest that slope‐dependent biomechanical responses observed in treadmill‐based laboratory studies may differ from those expressed during outdoor distance running, where pacing, physiological demands, fatigue, and terrain variability interact to influence running behaviour. Even moderate slopes, when repeated over long distances, can influence running mechanics. These observations highlight the importance of considering the interaction between slope, self‐selected velocity, and biomechanical loading in real‐world endurance running, although the directionality and underlying mechanisms of these relationships require further investigation.

## Funding

This research was supported by HKD $200,000 of internal funding from Hong Kong Baptist University.

## Ethics Statement

Ethical approval was obtained from the review board of Hong Kong Baptist University (REC/23‐24/0028/A5).

## Conflicts of Interest

Y.P. is a founder of Human Telemetrics (London), and founder of the original Sub2 marathon project now affiliated to Human Telemetrics (London, UK). The other authors declare that they have no conflicts of interest.

## Data Availability

The data that support the findings of this study are available from the corresponding author upon reasonable request.
